# [Corrigendum] miR-146a-5p protects against renal injury in MRL/lpr mice via improvement of the Treg/Th17 imbalance by targeting the TRAF6/NF-κB axis

**DOI:** 10.3892/etm.2026.13076

**Published:** 2026-01-28

**Authors:** Jiajia Teng, Feng Yang, Xiaoling Li

Exp Ther Med 25:21, 2023; DOI: 10.3892/etm.2022.11720

Subsequently to the publication of the above article, the authors have contacted the Editor to explain that, in Fig. 3 on p. 6, the data for the GAPDH band in Fig. 3D and the fluorescence images of the M146AG intervention group in Fig. 3G (the centre row) had been inadvertently selected incorrectly during image processing. After having comprehensively verified all the original datasets, the authors have corrected these errors by replacing the misplaced GAPDH band in Fig. 3D and the M146AG intervention group fluorescence images in Fig. 3G with the correct data, and the revised version of Fig. 3 is shown on the next page. The authors wish to confirm that these corrections do not alter the quantitative analyses, results or scientific conclusions of this study.

All the authors agree with the publication of this corrigendum; furthermore, they apologize to the Editor of *Experimental and Therapeutic Medicine* and to the readership for any inconvenience caused.

## Figures and Tables

**Figure 3:**
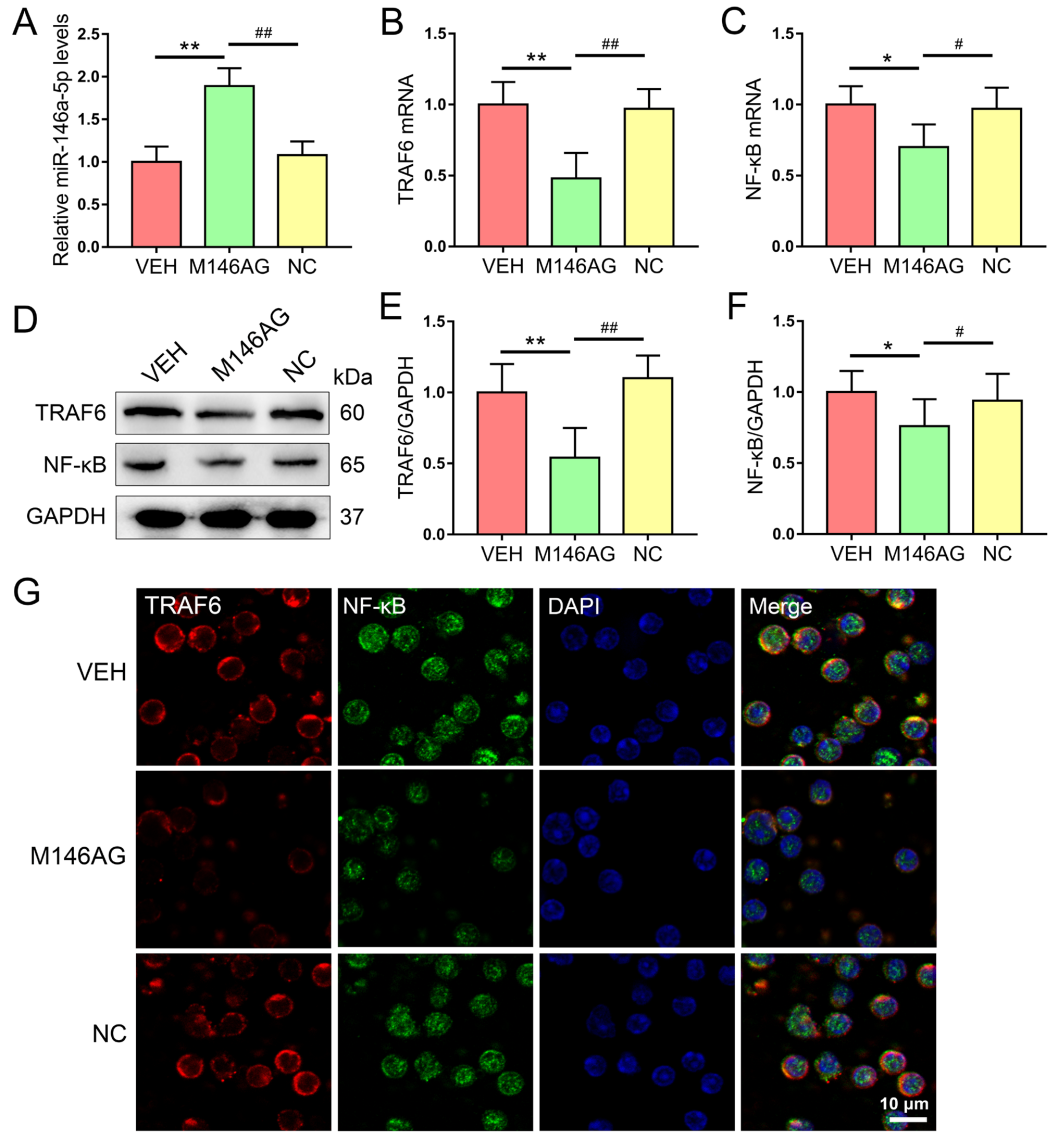
M146AG regulates expression of miR-146a-5p and TRAF6/NF-κB axis components in CD4^+^ T cells of MRL/lpr mice. (A) Relative miRNA expression level of miR-146a-5p. The relative mRNA expression levels of (B) TRAF6 and (C) NF-κB. (D) Representative western blotting bands of TRAF6/NF-κB axis protein expression levels. Semi-quantified relative protein expression levels of (E) TRAF6 and (F) NF-κB. (G) Protein expression levels of TRAF6 and NF-κB were assessed using immunofluorescence staining (scale bar, 10 µm). All data are presented as the mean ± standard deviation. ^*^P<0.05 and ^**^P<0.01. VEH, vehicle; NC, negative control; miR, microRNA; M146AG, miR-146a-5p agomir; TRAF6, tumor necrosis factor receptor-associated factor 6.

